# Social isolation and group size are associated with divergent gene expression in the brain of ant queens

**DOI:** 10.1111/gbb.12758

**Published:** 2021-06-20

**Authors:** Fabio Manfredini, Carlos Martinez‐Ruiz, Yannick Wurm, De Wayne Shoemaker, Mark J. F. Brown

**Affiliations:** ^1^ School of Biological Sciences University of Aberdeen Aberdeen UK; ^2^ School of Biological Sciences Royal Holloway University of London Egham UK; ^3^ School of Biological and Chemical Sciences Queen Mary University of London London UK; ^4^ Department of Entomology and Plant Pathology The University of Tennessee Institute of Agriculture Knoxville Tennessee USA

**Keywords:** colony founding, fire ants, gene expression, group size, isolation, social behavior, *Solenopsis invicta*

## Abstract

Social life and isolation pose a complex suite of challenges to organisms prompting significant changes in neural state. However, plasticity in how brains respond to social challenges remains largely unexplored. The fire ants *Solenopsis invicta* provide an ideal scenario for examining this. Fire ant queens may found colonies individually or in groups of up to 30 queens, depending on key factors such as density of newly mated queens and availability of nesting sites. We artificially manipulated availability of nesting sites to test how the brain responds to social versus solitary colony founding at two key timepoints (early vs. late colony founding) and to group size (large vs. small groups). We adopted a powerful neurogenomic approach to identify even subtle differences of gene expression between treatment groups, and we built a global gene co‐expression network of the fire ant brain to identify gene modules specifically associated with the different components of the social environment. The difference between group and single founding queens involves only one gene when founding behavior is still plastic and queens can switch from one modality to another, while hundreds of genes are involved later in the process, when behaviors have lost the initial plasticity and are more canalized. Furthermore, we find that large groups are associated with greater changes in gene expression than small groups, showing that even potentially subtle differences in the social environment can be linked to different neurogenomic states.

## Graphical abstract

Exposure to social isolation and varying group size are associated with significant changes in the levels of expression of genes in the brain of fire ant queens.

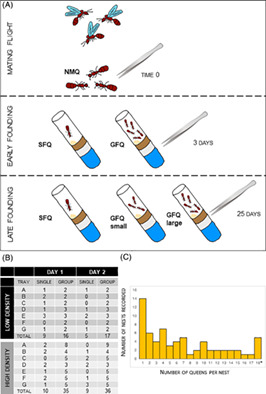

## 1 INTRODUCTION

The social environment is a major force at play in animal groups and is tightly linked to a broad range of phenotypic traits at both the structural and functional levels,^1^ including brain size,^2^, ^3^ brain anatomy,^4^, ^5^, ^6^ and brain gene expression.^7^, ^8^, ^9^, ^10^ One key feature of the social environment is group size,^11^ as, in principle, larger animal groups offer the possibility for a broader range of interactions among individuals.^12^ However, other factors play a key role within the social environment, such as dominance hierarchies, reproductive skew, numbers of breeders and division of labor,^13^ and it is often challenging, therefore, to assess how group size influences the social environment of an organism. Furthermore, it is not clear how groups of size equal to one (social isolation) should be compared with respect to large and small social groups. In principle, isolation is at the opposite end of the social spectrum compared with large social groups, and therefore should have very small impact on those phenotypic traits that are normally associated with life in social groups. Nevertheless, social isolation can trigger very powerful responses at multiple levels, including neurogenesis,^14^, ^15^ gene expression,^16^ and overall physiology and behavior,^17^ which are similar to what has been reported for complex social environments.

An open question in the field is whether exposure to social groups of different size is linked to varying levels of brain capacity within the same species. It is known, for example, that rearing‐group size during development can shape brain structure and functions in multiple ways.^18^ However, within‐species social groups are often unstable, and hence the behaviors displayed are characterized by high levels of plasticity.^19^, ^20^ Brain size is normally a good proxy for the number of neurons and the extent of the connections among them (Ref. 21 for a full overview on this relationship). However, simple measures of brain size do not take into account how neurons function,^22^ for example, within neural circuits.^23^ One way to approach this is to characterize the brain at a molecular level, to see for example, whether the transcriptional activity of neuronal genes or key regulators of brain functions change according to exposure to groups of different size or to social isolation.^24^

Colony founding in fire ants represents an ideal scenario to address these questions. Newly mated queens of *Solenopsis invicta* can experience two drastically different social environments when setting up a new colony: total isolation, when a single queen relies exclusively on her own resources to produce the first generation of workers, or group‐founding, when multiple queens share the same nest.^25^ In this second scenario, social groups can be of different size (from 2 to ~30) and provide the opportunity to explore the different social dynamics associated with small versus large groups. Furthermore, colony founding in *S. invicta* is a dynamic process, characterized by (a) high plasticity at initiation, when queens normally move from nest to nest and can shift between single and group‐founding strategies^26^, ^27^; (b) a subsequent more stable phase of approximately 3–4 weeks, when queens seal themselves in the nesting chamber and adhere to the founding modalities they have opted for (single or group) until the emergence of the first workers; and (c) a dramatic “conflict phase” in group‐founding queens, that kicks in after worker emergence and terminates with the survival of only one queen in the colony, while all the others either leave the nest or are executed.^28^, ^29^, ^30^ Newly mated queens from the same ant population (and even from the same nest) can adopt either of the two modalities of colony founding. The “choice” appears to be influenced purely by ecological factors, such as the density of newly mated queens within a certain area, and the availability of nesting sites.^25^ In fact, there is no known genetic pre‐condition, such as variation at specific loci, that determines whether a newly mated queen will adopt the single or group founding modality.

Here we used a powerful transcriptomic approach, characterized by high sequencing depth (42 million reads per sample on average), good biological replication and multiple time‐points, to explore global patterns of gene expression in the brains of *S. invicta* queens exposed to different social environments. We hypothesized that differences in the social environment present different behavioral challenges to queens that can be quantified through the measure of differential gene expression in their brain. A growing body of research is showing the potential of this approach in a wide range of social insects.^31^, ^32^, ^33^, ^34^ We analyzed group‐founding and single‐founding queens in relation to queens that had just returned from a mating flight, to explore how gene expression changes as a result of exposure to the two drastically different social environments. Furthermore, we examined the impact of more subtle differences in the social environment, by performing a comparative analysis of large and small groups (i.e., 8–21 vs. 2–6 queens per group, respectively), to characterize gene expression patterns associated with variable group size. As large and small groups are formed by founding queens from the same population and experience the same social dynamics (e.g., proportions of breeders or ranges of social ranks within the group), we assumed that group size (and not other social dynamics that could be associated with either social environment) would be the major correlate for differential gene expression in the brain. Finally, in our comparative analysis of single and group founding queens we considered two timepoints, to understand how brain gene expression changes in association with different levels of behavioral plasticity. Specifically, we sampled queens at an early stage (3 days post‐mating flight), when the modality of colony founding is still very plastic,^35^ and compared them to queens from a period when founding behavior is fixed. This was identified as 25 days post‐mating flight, when workers have not emerged yet and groups are stable,^28^, ^36^ that is, no openly aggressive interactions are detectable and all queens in a group are visible within the nest chamber, next to the eggs pile.

## 2 MATERIALS AND METHODS

### 2.1 Sample collection and housing

Newly mated queens of *S. invicta* were sampled on 4 May 2014 in a parking lot in Gainesville (Florida, coordinates 29.6220°N, 82.3838°W) immediately after a big mating flight. This area is densely populated by monogyne colonies, as reported in the literature.^37^, ^38^, ^39^ Queens were individually collected with forceps directly from the tarmac and transferred to a small plastic cup (Figure S1). All these queens were wingless, hence they had spent several minutes up to 2 h on the tarmac looking for a suitable nesting site. In fact, within 2 h from a mating flight all queens usually disappear from above ground in field observations.^35^ A set of 34 queens was frozen on dry ice immediately after collection in the field. These are the newly mated queens group (from now on NMQ), which represents the baseline for gene expression analyses in this study.

**FIGURE 1:**
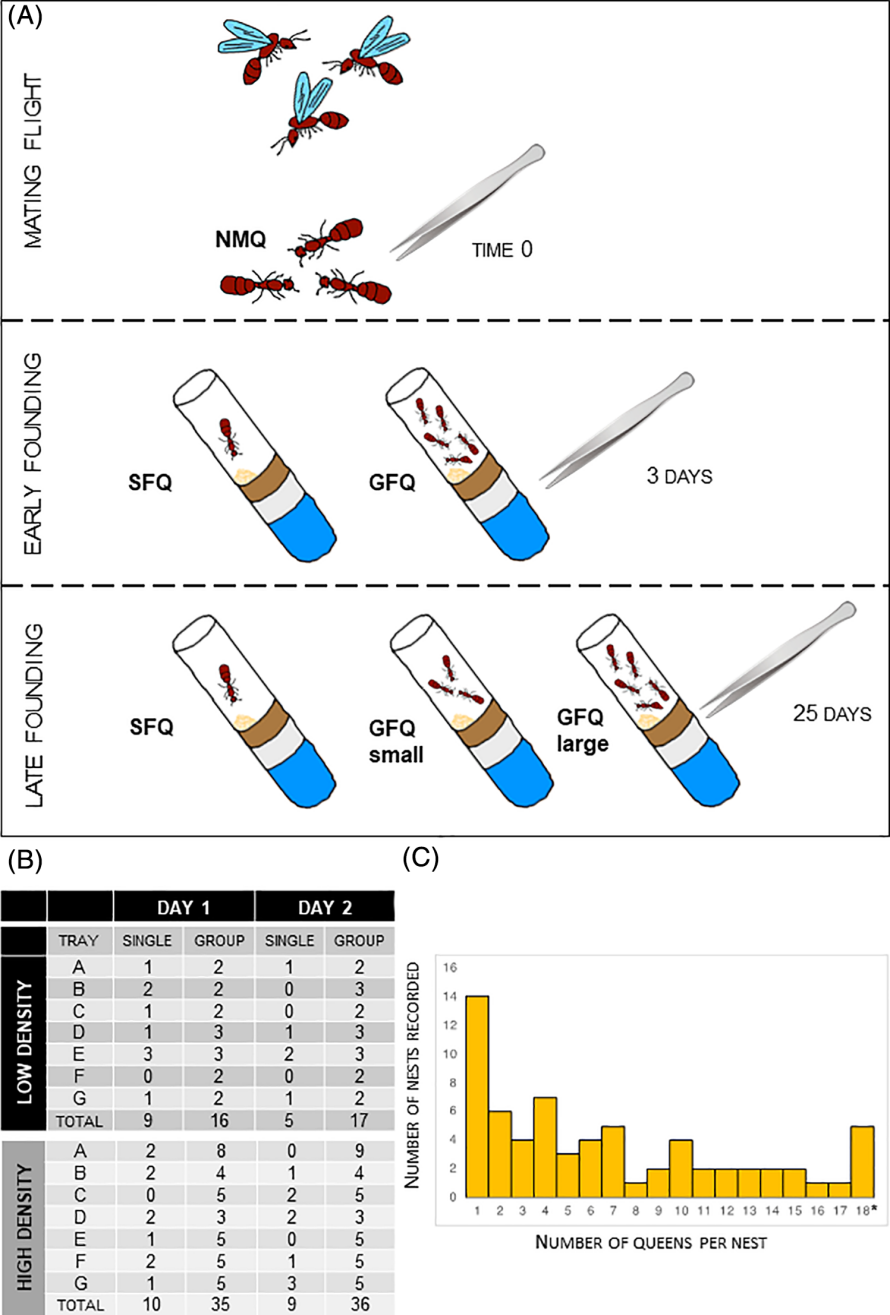
Experimental setup and sample collections. (A) Queens were sampled after a mating flight and reared in artificial nesting chambers in the lab. Focal queens were frozen at three key time points for RNA sequencing: 0, 3 and 25 days post‐founding. (B) Numbers of individual queens and associations that were recorded during the first 2 days of the process when different availabilities of nesting sites were simulated: large trays with abundant nests (“low density” of queens) or small trays with fewer nests (“high density” of queens). (C) Proportions of individual queens and groups of different size that were observed at day 2 post‐founding. Note: This category includes founding groups of 18 or more queens (maximum recorded = 30). GFQ, group‐founding queens from small (2–6 queens) and large groups (8–21 queens); NMQ, newly mated queens; SFQ, single‐founding queens

The other queens were setup to adopt one of two modalities of colony founding that are both recurrent in populations of *S. invicta* in the United States^25^: single‐founding (SF, 1 queen per nest, also called “haplometrosis”) or group‐founding (GF, ≥2 queens per nest, also called “pleometrosis”). After a set of plastic cups (12 total) was completed, the queens were released in large trays containing nesting chambers (Figure S2) where fire ant queens usually build their colony in lab conditions. As the mode of colony founding in the field is density dependent (i.e., group‐founding is more frequent when the rate of queen‐queen encounters is higher^25^), we used two different setups to promote spontaneous formation of SF and GF nests. We used lower density to promote SF: this consisted of releasing 24 queens in a large tray containing 24 nesting chambers (7 trays total). Conversely, we used higher density to promote GF associations: here 48 queens were released in a smaller tray containing only 14 nesting chambers (7 trays total). Ultimately, the proportion of nests that were SF was slightly higher for low‐density groups as we expected (1/3 of the total vs. 1/4, Figure 1(B)).

All 14 trays were transported to an environmental chamber where queens were reared in standard claustral conditions (no food, no water, in the dark). For the first 2 days, nesting chambers were left open to allow queens to move from one chamber to another if they wanted (mimicking what normally happens in the field). We recorded the numbers of SF and GF nests for both days (Figure 1(B)). At the end of day 2, a good mix of different options for colony founding was reached, with many SF nests (N = 14, 21% of the total) and a large proportion of GF nests (N = 53, 79% of the total) covering a wide range of group sizes (from 2 to 30, Figure 1(C)). We transferred each nesting chamber to a separate pencil box: from this moment queens were no longer allowed to move across nests, reproducing what usually happens in the field, when queens seal themselves into their nesting chamber and never leave it again. We kept queens in these conditions—claustral colony founding^40^—until the final sampling at 25 days post‐founding. We monitored incipient colonies on a daily basis to check that no workers had emerged in nests where we sampled queens, and also that colonies were peaceful (i.e., no evident aggression) in nests where we sampled group‐finding queens. Although detailed observations have not being carried out on social interactions among group‐founding queens, normally the occurrence of aggressive interactions can be easily directly spotted multiple times per day and also indirectly inferred when one or multiple queens are seen outside the nesting chamber.^36^

### 2.2 Experimental design

Prior to allocating queens to experimental groups for RNA sequencing (RNAseq) we dissected abdomens to check the spermatheca for mating status and to look at ovary development. In fire ants, the spermatheca is easily visible upon dissections in the abdomens of mated queens, where it appears as a bean‐shaped white structure filled with sperm (Figure S3(C)); when queens are unmated this structure is significantly less conspicuous and very hard to recognize, as it is tiny and transparent and it blends with the other abdominal tissues. Only mated queens (visible spermatheca) that had fully developed eggs visible within their ovaries (Figure S3(B)) were considered for this study. This step was performed to avoid any confounding effect of mating and reproductive status of queens on brain gene expression, as our aim was to focus specifically on gene expression associated with founding behavior and type of social environment.

We used NMQ (N = 6) as a control group with baseline gene expression levels to compare against both time points that were analyzed; NMQ were randomly picked from the pool of queens that were frozen immediately after collection, after confirmation of their mating status. For the earlier stage of the founding process, that is, 3 days, we compared the following two groups of queens against NMQ: (a) single‐founding queens at 3 days post‐founding (SFQ 3dpf, N = 6); and (b) group‐founding queens at 3 days post‐founding (range 12–30 queens per group, see Dataset S1 for details, GFQ 3dpf, N = 6). For the later stage of the founding process, that is, 25 days, we compared the following three groups of queens against NMQ: (a) single‐founding queens sampled at 25 days post‐founding (SFQ 25dpf, N = 5); (b) group‐founding queens at 25 days post‐founding from small groups (range 2–6 queens per group, see Dataset S1 for details, GFQsmall 25dpf, N = 5); and (c) group‐founding queens at 25 days post‐founding from large groups (range 8–21 queens per group, see Dataset S2 for details, GFQlarge 25dpf, N = 5). Despite some similarity in size at the 25dpf time of sampling for some of the groups that belonged to the two different categories (e.g., GFQlarge of size = 8 vs. GFQsmall of size = 6) it is important to note that all large groups started from group size ≥17 and progressively shrank in size because of the queen mortality that naturally occurs among fire ant foundresses (Figure S10); small groups instead started from size ≤6, therefore the size difference between the two categories was significantly higher across a large portion of the founding process than it might appear. SFQ 25dpf were obtained from seven initial GF associations (range 7–11 queens per group) on day 4 post‐founding. All queens from these associations were relocated to a new nesting chamber housed in an independent pencil box. This step was performed to start from a more homogeneous cohort of queens so that any difference in brain gene expression at 25dpf could be clearly linked to the fact that some queens spent 22 days in isolation versus being in a small or large group. To avoid pseudoreplication every queen analyzed for one of the GFQ treatments came from a unique founding group, that is, no founding group was represented more than once in our experimental design. No workers had emerged in any of the experimental colonies at the time of queen sampling.

### 2.3 Molecular work and statistical analysis of gene expression

All queens were flash frozen on dry ice and immediately transferred to a −80°C freezer for later processing. We placed individual heads on dry ice, we exposed the brain by gently scraping off the cuticle and other off‐target layers (e.g., frozen hemolymph), and we removed both eyes, mouthparts and associated glands.

We isolated total RNA from individual brains as described in the Appendix S1. We aimed to include only samples with total RNA > 200 ng (based on a NanoDrop™ spectrophotometer instrument, ThermoFisher) and RIN value ≥7 (TapeStation System, Agilent Technologies) in the RNAseq experiment. However, because of limitation in the number of replicates, we included two samples that had RIN value between 6 and 7 (see Dataset S1 for full details on all samples included in the study). Subsequent steps were performed by Beckman Coulter Genomic (now GENEWIZ) at their facility in the United States: this included cDNA synthesis, library preparation using the Illumina TruSeq Stranded Total RNA with Ribo‐Zero Kit, and sequencing on an Illumina HiSeq 2500 platform.

RNAseq read files were aligned to the *S. invicta* genome (assembly gnG, release 100 from refSeq) using the intron‐aware STAR aligner, version 2.6.1a.^41^ Estimated read counts were obtained with Kallisto and used to perform analyses of gene expression with DESeq2 (see supporting information for a full description of these analyses). We also performed Gene Ontology analyses using DAVID Bioinformatics Resources 6.8,^42^ weighed gene‐coexpression network analysis using the R package WGCNA,^43^ version 1.68, and gene enrichment analyses in R (see Appendix S1). We discuss the output of gene expression analyses focusing on individual genes when the output of pairwise comparisons was small enough to allow it; otherwise we adopt a broader approach and discuss GO terms when differences were larger. For network analyses, we focus on modules that show significant association with a trait of interest and discuss individual genes only for modules of smaller size.

## 3 RESULTS AND DISCUSSION

### 3.1 Expression profiles of grouped and single queens progressively diverge over time

**FIGURE 2:**
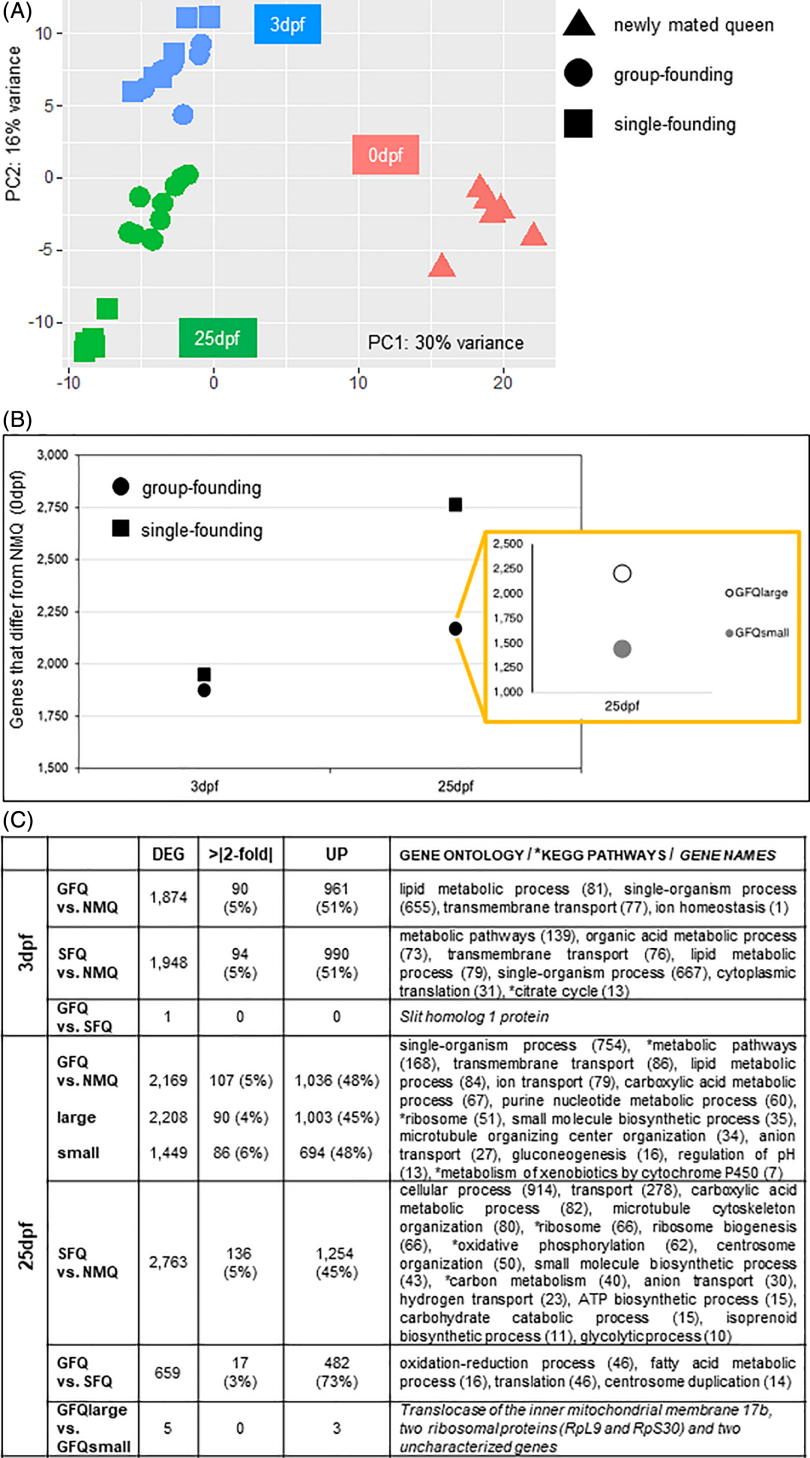
Gene expression analyses of group‐founding versus single‐founding queens. (A) Principal Component Analysis of all queen samples included in this study. The first component (30%) explains the difference between newly mated queens and all other groups of queens, while the second component (16%) explains the difference between the two time‐points of collection for founding queens, that is, 3 and 25 days post‐founding (3dpf and 25dpf, respectively). (B) Number of gene differentially expressed (FDR < 0.001) in group‐founding queens and single‐founding queens at 3 and 25 days post‐founding. The inset shows the details of large and small groups at 25 days post‐founding. Differentially expressed genes for all groups are calculated with respect to newly mated queens at time 0. (C) Summary table for gene expression data produced by all pairwise comparisons of interest. Only gene ontology terms and KEGG pathways that survived Benjamini correction (*P*‐value < 0.05) are reported or, when only few genes were differentially expressed, genes names are indicated. DEG, significantly differentially expressed genes; dpf, days post‐founding; GFQ, group‐founding queens from small (2–6 queens) and large groups (8–21 queens); NMQ, newly mated queens, SFQ, single‐founding queens; UP, upregulated

We performed a series of analyses to explore whether group‐founding queens (GFQ) differ from single‐founding queens (SFQ) in their overall neurogenomic state (Figure 1). Both groups of queens significantly differed from NMQ (the baseline or control group for brain gene expression in this study) at both the early and late founding stages: PCA analysis showed that 30% of global gene expression can be explained by differences between NMQ and all other queens (Figure 2(A), Figures S12–S15 and Dataset S5). In line with this, hierarchical clustering analyses showed that NMQ are the outgroup in both analyses (Figure S4). This clearly indicates that founding behavior per se is the major factor that correlates with a queen's neurogenomic state, while social environment associated with modality of colony founding and group size are secondary factors. We also detected a general pattern of increased differential expression over time in both groups of queens compared with NMQ; however, SFQ displayed a higher proportion of genes that were statistically different from NMQ than GFQ (Figure 2(B)). To understand this pattern, we examined the difference between the two groups of queens and NMQ separately for each time point.

At 3dpf, both GFQ and SFQ differed from NMQ for similar numbers of genes, that is, 1874 and 1948, respectively: the two sets both represent 13% of the total and are not significantly different in size (X^2^ test from equal: X^2^ = 0.72, df = 1, *P =* 0.40). The two sets also showed very similar proportions of genes that were more highly expressed and with expression levels more than 2‐fold higher compared with NMQ (Figure 2(C)). Finally, they largely overlapped: 1431 of the significantly differentially expressed genes (>73% of the genes in either group) were shared across the two groups, a 5.7‐fold higher proportion than expected by chance (hypergeometric test: *P <* 0.001). These genes are likely involved in the general patterns associated with founding per se and onset of reproduction, that all queens shared at this stage, and therefore are less relevant for our study. These results clearly indicate that the difference between the neurogenomic states of GFQ and SFQ is minimal at 3dpf. This was supported by the fact that only one gene was significantly different between GFQ and SFQ at this time point when we compared them directly (see Figure 2(C) and further details below). We suggest that the minimal difference in gene expression at 3dpf might be linked to the behavioral plasticity of founding queens at this stage, when they often move from nest to nest and possibly switch across GFQ and SFQ modalities (Figure 1(B) and Ref. 25).

Later in the founding process the scenario changed dramatically. In fact, at 25dpf, there were 2169 genes in the brain (15% of the total) whose expression was significantly different between GFQ and NMQ, while this was the case for 2763 genes (19% of the total) in SFQ: the difference between the sizes of the two gene sets is statistically significant (X^2^ test from equal: X^2^ = 35.90, df = 1, *P <* 0.01). Despite being different in size, the two gene sets largely overlapped, similarly to what was reported for 3dpf: 1614 of the significantly differentially expressed genes were shared across the two groups, a 3.9‐fold higher proportion than expected by chance (hypergeometric test: *P <* 0.001). These common genes are likely associated with the general biological processes that all queens experience at this stage, such as egg‐laying, brood care and aging.

The main result of the comparison between GFQ and SFQ holds even if we consider GFQlarge and GFQsmall separately (to keep sample size constant across groups, N = 5): both the 2208 genes that significantly differed between GFQlarge and NMQ, and the 1449 genes that significantly differed between GFQsmall and NMQ were smaller than the 2763 genes that significantly differed between SFQ and NMQ (X^2^ test from equal: X^2^ = 31.07 and X^2^ = 210.07, respectively, df = 1, *P <* 0.01). The two sets showed similar proportions of genes that were more highly expressed compared with NMQ and also the same proportion of genes with large fold changes compared with NMQ (Figure 2(C)). PCA analysis supported the clear separation between GFQ and SFQ at 25dpf (Figure 2(A), Figures S12–S15 and Dataset S5). It is clear that, at this stage of the founding process, the social environment that the queens experience affects their neurogenomic state to a larger extent than at 3dpf. This reflects their social history, with SFQ having spent 25 days in total isolation while GFQ were surrounded by a network of social interactions with nestmate queens. Furthermore, it is important to note that at 25dpf the fate of the two groups of queens also drastically diverges: SFQ no longer accept additional queens in the nest (they will aggressively reject them), while GFQ persist as social groups, which will transition to a phase of conflict later in the process of colony founding that will precipitate after the emergence of the first workers in the nest.^28^ Therefore, it is possible that brain gene expression is being re‐programmed towards two different directions at this point: towards a linear monogyne social form of colony life in SFQ (one queen per colony) versus more social dynamics (and conflict) in GFQ before monogyny is eventually reached.

### 3.2 Specific brain gene sets exhibit differential expression in response to both isolation and prolonged exposure to social environments

We performed a second set of analyses to directly compare GFQ and SFQ and identify groups of genes that are significantly associated with group living versus isolation. First, we built a global gene expression network, encompassing all 33 queens used for this study, and we identified network modules (groups of genes) that were significantly associated with GFQ or SFQ. Second, we performed direct pairwise comparisons between GFQ and SFQ at 3dpf and 25dpf, to characterize the key genes that were significantly differentially expressed in the two groups of queens at the two time points.

#### 3.2.1 Global gene network and module‐trait association analyses

**FIGURE 3:**
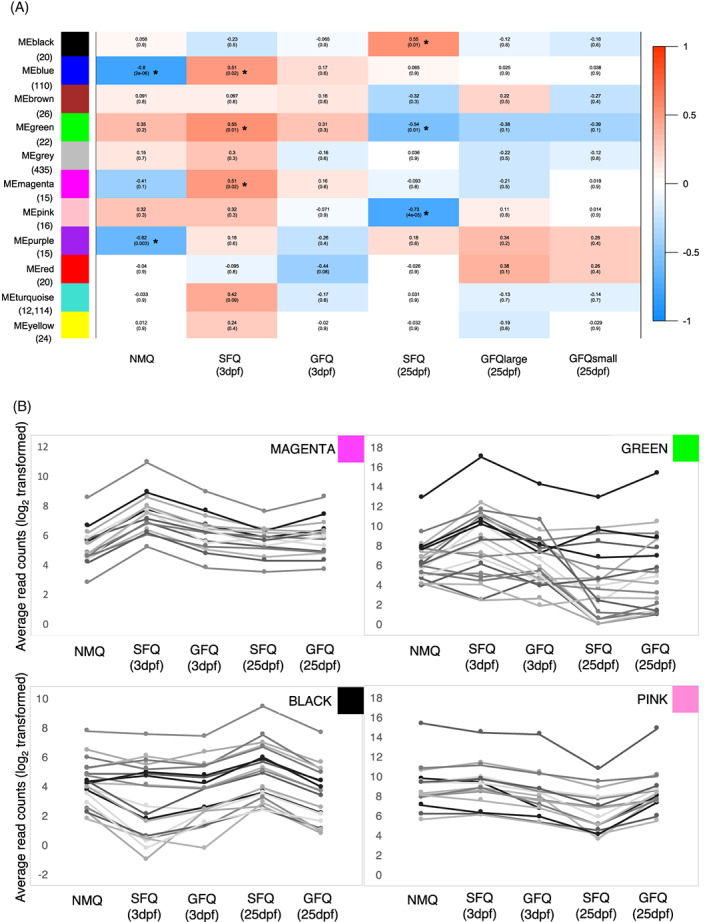
Weighed gene‐coexpression network analysis. (A) Module‐trait association analysis, showing what modules are significantly associated (*) with each group of queens. The matrix is color coded, with warm colors indicating positive associations (*x*‐value > 0) and cold colors indicating negative associations. *P*‐values corrected for multiple testing (Benjamini‐Hochberg) are also indicated below. Below each module, in brackets, is indicated the total number of genes within the module. (B) Details of the patterns of expression in five groups of queens for all the genes included in four modules (magenta, green, black, and pink) that were significantly associated with single‐founding behavior. Raw data for the genes in each module are available in Dataset S4. dpf, days post‐founding; GFQ, group‐founding queens from small (2–6 queens) and large groups (8–21 queens); ME, module eigenvalue; NMQ, newly mated queens; SFQ, single‐founding queens

The fire ant brain gene network encompassed 11 modules (Figure 3(A)), ranging in size from small (15 genes in the magenta and purple modules) to very large (12,114 genes in the turquoise module). No network modules were significantly associated with GFQ (FDR > 0.05), whereas five modules were associated with SFQ (Figure 3(A) and Dataset S4). Two modules (blue = 110 genes and magenta = 15 genes) were positively associated with SFQ at 3dpf, hence they represent sets of genes that quickly respond to early social isolation. Intriguingly, 10 of the 15 genes in the magenta module matched predicted *S. invicta* G‐protein coupled receptors (key receptors of brain neural cells^44^) in the *methuselah* cluster (*Mth‐like*, *Mth‐like 3*, and *Mth2‐like*), a group of genes known to extend lifespan in *Drosophila* when less expressed.^45^, ^46^ There are nine Mth‐like receptors in *S. invicta*,^47^ and four of these (*Mth‐like 1*, *3*, *5*, and *10*) showed significantly differential expression between single‐founding and pair‐founding queens 1 month after colony founding in a previous microarray study^36^: *Mth‐like 10* was more highly expressed in single‐founding queens, while the other three were more highly expressed in pair‐founding queens. Our study supports the idea that social environment and aging are tightly linked in fire ant founding queens, and shows that the interaction is particularly evident in SFQ very early in the founding process, probably as a response to isolation.

Two modules (black = 20 genes and pink = 16 genes) were both associated with SFQ at 25dpf but in opposite directions: therefore, they both represent sets of genes that respond to long‐term exposure to social isolation, but follow opposite patterns of expression (Figure 3(A,B)). Several vision‐related genes were included in this group: *ninaA* (LOC105194667)*, ninaC* (LOC105200050), *Arr1* (LOC105199319), and *Arr2* (LOC105202669) all showed patterns of upregulation in SFQ at 25dpf (black module). Interestingly, the regulation of vision‐related genes has been observed in other insects following mating and it has been linked to the switch from photophilic to photophobic behavior.^34^, ^48^, ^49^ Unfortunately, this does not explain, however, why vision‐related genes were expressed at higher levels in SFQ only and not in GFQ, which also underwent a similar process of ground‐nesting behavior after mating. Finally, *Lsp1beta* (LOC105192919, pink module) was less expressed in SFQ at 25dpf. This gene is a close relative of *Lsp2*, involved in synapse formation in *Drosophila* ^50^ and it was more highly expressed in aggressive queens within founding pairs of the ant *Pogonomyrmex californicus*.^51^

A fifth network module (green = 22 genes) showed opposite patterns in SFQ at the two time points: in fact, it was positively associated with SFQ at 3dpf and negatively associated with SFQ at 25dpf (Figure 3(A,B)). Hence, this small set of genes may play a role in the transition from incipient colony founding to colony establishment in SFQ, and might be responsible for the progressive canalization of gene expression that accompanies the loss of behavioral plasticity in SFQ as a consequence of social isolation. There was only one gene in the green module with known function in *Drosophila*: *yolkless* (LOC105200757), encoding the Vitellogenin receptor. Vitellogenin is an important reproductive protein in insects, responsible for the formation of the egg yolk,^52^ but recent studies have linked the expression of *vitellogenin* in the insect head and brain to important social behaviors, like parental care or social aggression^53^, ^54^, ^55^ and it is hypothesized that ant *vitellogenins* and *Vg‐like* genes might have expanded their functional repertoire following major duplication events.^56^ If *vitellogenin* plays a role in the regulation of DNA functions in the insect brain, its expression in isolated queens could be the key mechanism of their behavioral response to social isolation. We looked at the expression patterns of the two *S. invicta vitellogenins* (*Vg2* LOC105205782 and *Vg3* LOC105205783) that are known to be preferentially expressed in queens.^57^ Interestingly, both genes were more highly expressed in queen foundresses compared with NMQ at 3dpf, while only *Vg3* followed this pattern also at 25dpf; no difference was observed between GFQ and SFQ (Datasets S2 and S3). These observations seem to suggest that brain expression of *vitellogenins* in fire ant queens is more linked to colony founding *per se* or reproductive behavior rather than response to social environment. In addition, a group of genes in the green module are associated with chemical communication: the two putative odorant receptors *Or71a* and *Or22c* (LOC105206746 and LOC105206770, respectively), and three predicted odorant binding proteins (SiOBP3 LOC105194481; SiOBP4 LOC105194487; and SiOBP13 LOC105194495). Finding that odorant receptors are expressed in an insect brain is puzzling, as expression of these genes is normally localized on sensory organs.^58^ However, while we note that the overall levels of expression of *Or71a* and *Or22c*, although consistent across all queens analyzed, were rather low in our experiments (less than 10 reads per sample on average), brain expression of ORs has been reported before in social insects (e.g., Refs. 49, 59). Further studies are needed to understand the origin and function of these expression patterns. Intriguingly, all three OBPs identified in the green module are located in the social chromosome “supergene” region that determines whether established colonies of *S. invicta* accept multiple queens.^60^, ^61^ This prompted us to investigate whether genes in the supergene (640 genes out of a total of 14613 genes in the fire ant genome) were overrepresented in the green module. This was the case for 6 of the 22 genes in the module, which is more than expected by chance (Fisher test, *P =* 0.002 after correction for multiple testing, Figure S5). This supports the idea that genes in the supergene region play important roles in shaping a queen's reaction to her social environment. It is tempting to speculate, for example, that the variation in expression of such genes could affect the production or perception of odors of other queens within the nest.

#### 3.2.2 Pairwise comparisons of gene expression

Expression of only 1 gene was significantly different between GFQ and SFQ at 3dpf and FDR < 0.001: *Slit homolog 1 protein* (LOC105202267, 1.3 times higher in SFQ). *Slit* is associated with axon guidance, dendrite morphogenesis, and neuron differentiation and migration in *Drosophila*.^62^ In the context of founding behavior in fire ant queens, the fact that *Slit* is the one gene that differs between GFQ and SFQ (being more highly expressed in SFQ) suggests that future studies should explore its role in the process of brain restructuring caused by the lack of social interactions during isolation.

A much larger difference between GFQ and SFQ was observed at 25dpf, when 659 genes (4.5% of the total) significantly differed at FDR < 0.001 (Figure 2(C)). A large proportion of these genes (75%) was more highly expressed in GFQ, indicating that at this stage in the founding process life in social groups correlates with higher transcriptional activity of genes. As these measures were performed in the brain specifically, we hypothesize that group‐living triggers higher neural response in fire ant queens than isolation, although targeted functional tests (e.g., artificial manipulation of the social environment) are needed to support a causal link between exposure to social interactions and increased neural activity in the brain. Interestingly, a study in guppies showed that exposure to a group of conspecifics activated a specific region of the forebrain when compared with social isolation.^63^ This activation, measured as increased expression of an immediate early gene (*egr‐1*), was explained as a stimulation of the reward system in the fish brain because of the sight of conspecifics. A similar mechanism could be in place for GFQ in our study or, alternatively, increased gene expression could be explained by a release of inhibition in the regulation of large group of genes because of repeated social stimulation by nestmates. Further studies are needed in the future to test which, if any, of these hypotheses holds true.

The difference between GFQ and SFQ at 25dpf is also in line with a previous microarray study, where a large set of genes significantly differed between single‐founding queens and pair‐founding queens sampled at a later stage in the founding process, when the conflict phase had already started among paired queens (3192 genes at FDR < 0.001 or 34% of the total analyzed^36^). Aging is the most interesting process that was significantly overrepresented among genes that differed between GFQ and SFQ in our study (GO analyses, Dataset S3). Some of the genes in this group were also found in the microarray study,^36^ such as *I'm not dead yet* (LOC105193770), the superoxide dismutase genes *Sod* (LOC105208009) and *Sod2* (LOC105203964), and the peroxiredoxin genes *Prx3* (LOC105205792) and *Prx5* (LOC105195487), similar to *peroxiredoxins 6005* and *5037* from the microarray study. The fact that the same longevity genes also respond to social environments in other species^64^, ^65^, ^66^ supports the hypothesis of a conserved function for these genes, which is also visible in fire ant queens. Here, the crosstalk between social environment and lifespan starts very early in the process of colony founding (3dpf) and continues for the whole duration, differentially affecting group‐founding and single‐founding queens. Aging most likely interacts with other physiological compartments that are differentially regulated in queen founders, for example, reproductive output, that might vary according to founding modality.^27^, ^67^ However, we did not detect any molecular signs for differential reproductive activation among queens in this study, in contrast to our previous microarray study where instead reproduction appeared as a major biological function affected by single versus pair founding.^36^ We attribute this discrepancy to the high specificity of the tissue samples analyzed in this study (brains vs. whole bodies in the microarray study) that are not suited to explore the regulation of reproductive functions. It remains unclear how exactly aging genes and the social environment influence each other, and also how these dynamics evolve after the first workers emerge and the founding process terminates.

We explored the hypothesis that genes in the supergene region were overrepresented among genes that were significantly differentially expressed between groups of queens. Of all pairwise comparisons, only GFQ versus SFQ at 25dpf was significantly enriched for such genes, no matter whether groups were large or small (KS test, *P <* 0.05, Table S1). These results are in line with the output of the network module‐trait association analysis, and further support the idea that the supergene region plays a role in discriminating SFQ queens from GFQ queens after prolonged exposure to social isolation.

### 3.3 Large social groups trigger bigger changes in brain gene expression than small groups

We compared gene expression in fire ant queens from large groups (GFQlarge, 8–21 queens per group) and small groups (GFQsmall, 2–6 queens) at 25dpf (Figure 1). GFQlarge queens differed from NMQs for a larger number of genes compared with GFQsmall (2208 and 1409, respectively, at FDR < 0.001, Figure 2(B,C)), indicating that life in larger social groups is associated with the regulation of a significantly larger proportion of genes in the brain (X^2^ test from equal: *X* ^2^ = 89.33, df = 1, *P <* 1e‐5). According to these observations, it seems that brain gene expression could be used as a proxy for estimating cognitive tasks associated with different social environments. Life in social groups of different size poses different cognitive challenges and it has been observed that members of large groups have more brain power, in particular when groups are stable (“social brain hypothesis”,^68^ but see Refs. 69, 70). On the other hand, levels of gene up‐regulation compared with NMQs were similar in the two groups of GFQs. This is in disagreement with what has been observed in primates, where significant up‐regulation of genes in the brain has been suggested as the driver for the higher cognitive functions observed in humans compared with other non‐human primates.^24^ This discrepancy could be because of the difference of comparing group size across different species (humans and other primates) versus within the same species (*S. invicta*).

A direct comparison of gene expression between GFQlarge and GFQsmall queens showed that only five genes were significantly different at FDR < 0.001 (Figure 2(C)): a translocase of the inner mitochondrial membrane, two ribosomal proteins and two genes of unknown function. A less stringent analysis (FDR < 0.05) identified 258 genes that were different between the two groups (see supporting information S1 for details on these genes).

## 4 CONCLUSIONS

Through a series of brain gene expression and gene network analyses we show that the neurogenomic state of an insect changes over time and in response to both drastic and subtle differences in the social environment. First, a major difference in the social environment (group living vs. isolation) is associated with significant proportions of genes that differ in their expression patterns. We show that this difference is minimal very early in the process of colony founding (only one gene at 3dpf), when fire ant queen behavior is typically plastic,^35^ but increases significantly once this plasticity is lost (hundreds of genes at 25dpf). Finally, a much subtler difference in the social environment (large vs. small social groups) is still visible at the level of brain gene expression, with larger groups associating with bigger changes in the neurogenomic state.

These results clearly illustrate the power and high resolution of the neurogenomic approach, making it an ideal complement to regularly adopted approaches such as the analysis of brain allometry (e.g., Refs. 5, 71) when investigating the effect of the social environment on individual organisms. There are also some evident limitations associated with transcriptomic studies overall, for example, the impossibility to establish causative links between traits of interest and gene expression. In this study, for example, we cannot exclude that other pre‐existing factors (e.g., differences in the DNA sequence at the gene level) might be driving differential gene expression in GFQ versus SFQ. In fact, we induced queens to opt for either modality of colony founding by manipulating queen density and availability of nesting chambers at the beginning of our experiment: it is possible that a queen's “choice” for one founding modality might have been dictated by some underlying conditions that we are unaware of. We opted against arbitrarily assigning a founding modality as this would not be reflective of the complex dynamics that occur in the field, therefore precluding us from being able to uncover patterns of brain gene expression that are ecologically relevant. Also, we considered the fact that by presenting queens with the opportunity to switch colony founding modality, or even simply joining a different founding association, would provide the opportunity to explore gene expression patterns associated with behavioral plasticity more fully.

On the other hand, there are three considerations that advocate for the interpretation that social environment is driving gene expression in this study (rather than patterns being a consequence of pre‐existing genetic differences): first, we collected queens from a homogeneous population with low genetic diversity, as indicated by the population's history and genetic similarity as observed in previous years (see Methods) and also by the low rate of polyandry for fire ants colonies in the area^72^, suggesting that all queens from the same colony are genetically very similar; second, GFQ at 25dpf (the group that mostly differed from isolated queens) derived from initial SFQ (see Methods), hence the only difference between GFQ and SFQ at this time point reflected the time spent in social groups versus isolation; third, if there were pre‐existing factors that differed among groups of queens they had no effect on brain gene expression, as clearly shown by the detection of only one gene that was significantly differentially expressed between GFQ and SFQ at 3dpf. Clearly, we must also take into account that the gene expression analyzed here is just the end product of transcription and a range of other mechanisms could be responsible for the patterns that we see at the behavioral level: for example, different key regulators such as transcription factors or non‐coding RNA, not included in our analyses. The possible role of transcriptional regulatory elements and their integration with gene‐expression data surely deserves further investigation in the future (e.g., Luscombe et al. 2004).

It would be interesting in the future to further investigate the molecular basis for founding behavior in fire ants by comparing gene expression in different brain tissues, to test for example, whether more differences are observed in the mushroom bodies, the region associated with highly cognitive functions in insects,^73^ compared with the central complex or the optic and antennal lobes. Also, it would be interesting to look at GFQ at the end of the conflict phase, when all other nestmate queens have been eliminated, to see whether the brain can still display plasticity and transition back to an “isolation‐like” phenotype for its gene expression profiles, comparable to the profile of SFQ. This would be an excellent control experiment to also test whether queen age has any effect on the patterns of gene expression that we report in this study. In fact, we might expect that 3dpf queens are more similar to NMQs than 25dpf queens for age‐responsive genes. However, it is also possible that the short time‐span between the early and the late stage of colony founding might play very little role in the expression of age‐responsive genes, considering that fire ant queens can live for several years. Furthermore, it is important to note that testing gene expression in queens at a later stage would be problematic because of the presence of newly emerged workers in the colony, which necessarily triggers a radical change of the social environment. In conclusion, our results lay the ground for future research aimed at characterizing the genes and genome functions that regulate key animal behaviors like cooperative founding, group living and social isolation.

## CONFLICT OF INTEREST

The authors declare no conflict of interest.

## Supporting information


**Appendix S1**: Supporting information. Detailed methods for molecular work. Comparison between large vs. small groups of queens. Comparative network analyses.

**Dataset S1**: Sample details for RNAseq experiments. Details on ant queens selected for RNAseq, and quantities and quality of RNA samples from queens' brains that were used to prepare libraries. Only samples labeled in red were used in the end.

**Dataset S2**: Pairwise comparisons at 3dpf. Details are provided about gene expression analyses (fold‐change differences, p‐values and corrected p‐values for all genes analyzed) and Gene Ontology analyses (GO terms overrepresented at corrected p‐values<0.05) for all pairwise comparisons.

**Dataset S3**: Pairwise comparisons at 25dpf. Details are provided about gene expression analyses (fold‐change differences, p‐values and corrected p‐values for all genes analyzed) and Gene Ontology analyses (GO terms overrepresented at corrected p‐values<0.05) for all pairwise comparisons.

**Dataset S4**: WGCNA modules significantly associated with phenotypes of interest. Details are provided of the gene to gene correlation for all genes included in the module.

**Dataset S5**: Eigenvalues and loadings for all PCs obtained at the two timepoints.

## Data Availability

RNAseq data that support the findings of this study have been deposited in NCBI BioProject Submissions with the SubmissionID: SUB5134076s and BioProject ID: PRJNA525584 (www.ncbi.nlm.nih.gov/bioproject/?term=PRJNA525584). All codes used for the analyses described in the manuscript are publicly available on GitHub (github.com/FabioManfre/Solenopsis_RNAseq_founding‐queens).
