# Supplementary material for: Social isolation and group size are associated with divergent gene expression in the brain of ant queens
Source: Genes Brain Behav. 2021 Jun 20;21(3):e12758. doi: 10.1111/gbb.12758 (PMC9744527; doi:10.1111/gbb.12758)
Supplement: Supplementary file 1 — Appendix S1: Supporting information. Detailed methods for molecular work. Comparison between large vs. small groups of queens. Comparative network analyses. [file GBB-21-e12758-s003.docx]

Social isolation and group size are associated with divergent gene expression in the brain of ant queens

Fabio Manfredini^1,2*^, Carlos Martinez-Ruiz^3^, Yannick Wurm^3^, DeWayne Shoemaker^4^, Mark J. F. Brown^2^

**This PDF file includes:**

Detailed methods for molecular work

Comparison between large vs. small groups of queens

Comparative network analyses

Figures S1 to S15

Table S1

Legends for Datasets S1 to S5

**Other supporting information for this manuscript include the following:**

Datasets S1 to S5

**Detailed methods for molecular work**

Samples preparation. We placed individual brains in a plastic tube with QIAzol Lysis reagent (Qiagen, Manchester UK) and metal beads and disrupted brain tissues by means of a Tissue Lyser device (Qiagen). Total RNA was isolated using RNeasy Plus Universal kit (Qiagen) and following manufacturer instructions. RNA quantities and quality were checked with a Nanodrop instrument first and then with an Agilent Bioanalyzer. Samples that satisfied concentration and quality requirement were processed for RNA sequencing.

Analysis of gene expression. RNAseq read files were aligned to the *S. invicta* genome (assembly gnG, release 100 from refSeq) using the intron-aware STAR aligner, version 2.6.1a. We followed two approaches: we first used the official geneset for the fire ant genome deposited on NCBI (14466 genes), and second we also performed an alignment to an enhanced geneset obtained with Cufflinks, version 2.2.1^1^ (reference annotation based transcript assembly). This second approach allowed us to include in the reference geneset real genes missing from the official geneset, missing exons of previously known genes, or background transcription, for a total of 37326 predicted genes. The following steps were performed in parallel and we detected great overlap between the two approaches, in terms of overall patterns of gene expression (e.g. hierarchical clustering and PCA analyses) and also genes that were significantly differentially expressed between focal groups. The NCBI based approach was deemed more suitable for follow-up analyses, as the geneset associated with this approach had better annotations and therefore it allowed more in-depth GO analyses and characterization of genes of interest. To avoid confusion, in this manuscript we reported the output of the analyses associated with the NCBI based approach alone.

We imported the estimated read counts generated by Kallisto into R using Tximport v1.2.0^2^ and DESeq2 v1.24^3^. We quantified differential gene expression by combining two separate analyses for each of the two experiments: a likelihood ratio test to detect what genes were more differentially expressed across all levels (LRT approach); and planned pairwise comparisons between phenotypes of interest. We initially explored our dataset to detect any possible effect of rearing tray on brain gene expression in founding queens (Fig. S11). In all analyses, differential expression was obtained by correcting for multiple testing with FDR (false-discovery rate). We used the whole set of normalized count data processed with DESeq2 to perform Principal Component analyses, and we used the outputs of the LRT approaches to perform separate Hierarchical Clustering analyses for the two experiments. In this case, we used only the sets of genes that were differentially expressed at FDR<0.001 and we performed a clustering analysis based on queen phenotypes.

Gene Ontology analyses were performed using *Drosophila* orthologues for *S. invicta* genes. We performed Functional Annotation Clustering to group together related GO terms (Biological Processes only) or KEGG pathways, and we identify significantly enriched terms using a threshold of p-value<0.05 plus a correction factor using Benjamini=0.05.

Weighed gene-coexpression network analysis was performed on all 33 queens included in this study using the R package WGCNA, version 1.68, and with a soft-threshold power of 12. This value was chosen among a series of outputs as it was the closest to produce an R^2 value of 0.85 (normally recommended as an ideal target by the software developer in order to guarantee a scale-free network). Module-trait association analyses were performed in R, following the same approach as described in Ref.^4^. Briefly, we performed a correlation test using the “cor” function in R and we adopted a threshold for signification correlation of 0.05 after Benjamini-Hochberg correction. We correlated module eigengenes with “trait values” represented by each group of queens considered in this study, i.e. NMQ, SFQ 3dpf and 25dpf, GFQ 3dpf, GFQlarge and GFQsmall 25dpf. We also performed an additional WGCNA analysis where we compared two separate networks, one built on data from all GFQ and another one based on SFQ data. We are aware that sample size for this comparative analysis is limited as the number of individuals per network was below 20. We decide therefore not to include this analysis in the main text – but see section below for details on how this analysis was performed and relative outputs.

Gene enrichment analyses for elements of the supergene region were performed in R using two approaches. First, we performed an “enrichment per module” analysis, where we checked whether any of the modules obtained through the network analysis had more supergene loci than expected by chance. We used a Fisher test to verify whether the expected ratio of supergene elements was observed in each module based on what previously observed at the level of the whole genome – 640 genes in the supergene and 13973 outside^5^. As a second approach, we performed an “enrichment for differentially expressed genes” analysis, to test whether supergene elements were overrepresented among differentially expressed genes for each pairwise comparison. For this we used a Kolmogorov-Smirnoff test to compare the distribution of uncorrected P values between supergene and non-supergene loci per comparison.

**Comparison between large vs. small groups of queens**

A direct comparison of gene expression between GFQlarge and GFQsmall queens revealed that only 5 genes were significantly different at FDR<0.001. However, a less stringent analysis (FDR<0.05) identified 258 genes that were different between the two groups (Fig. S6). Many of these genes were significantly associated with behaviourally relevant functions (GO analyses, see supplementary tables) such as learning and memory with *dunce* (LOC105197141), *neuralized* (LOC105202574) and *Pka-C1* (LOC105193172), neural functions with *found in neurons* (LOC105193515), *foxo* (LOC105196024), *seven up*^6^ (LOC105207607) and *turtle* (LOC105207535), and regulation of mRNA processing.

The differential regulation of brain genes related to behavioural and neural functions supports the hypothesis that large groups differ from small groups in their cognitive demands, and also indicates that this difference might be associated with brain restructuring and protein synthesis leading to the formation of new synapses. Four genes in this group are of particular interest in the context of their potential role in the regulation of social behaviour in fire ant queens during colony founding (Fig. S7): *Src64B* (LOC105197191), *Dscam1* (LOC105203138), *Dscam2* (LOC105207772) and *SPARC* (LOC105197504). *Src64B* is a key regulator for the development of mushroom bodies, an important area in the insect brain responsible for the integration of a wide range of stimuli and usually associated with highly cognitive functions^7^. Levels of *Src64B* were 1.36 times higher in GFQsmall than in GFQlarge. Genes in the *Dscam* family are well known for their ability to generate many different protein isoforms through alternative splicing, and their function in the insect brain is linked to the formation of new neural connections^8,9^. Both *Dscam1* and *Dscam2* genes were more highly expressed in GFQsmall compared to GFQlarge (1.21 and 1.34 times higher, respectively). Finally, SPARC, known as the “gregarious specific gene” in locusts^10^ was more highly expressed in GFQsmall (1.81 times higher than in GFQlarge).

**Comparative network analysis of group-founding vs. single-founding queens**

Methods

Separate analyses of gene networks (WGCNA approach) were performed for the brains of all GFQ (N=16) and all SFQ (N=11) across the two time points, following the same approach as described in the main methods. We explored the overlap between the outputs of the two networks by performing an Alluvial Plot approach with the R package “ggalluvial” (version 0.10.0). For this we selected the set of genes that were clustered in modules in both GFQ and SFQ network analyses (excluding large turquoise and grey modules) and we visualize their clustering patterns across the two analyses.

As this comparative network analysis was based on groups of different samples size (16 vs. 11 queens, respectively), we performed an additional analysis to exclude that the higher number of modules found in GFQ was due to the larger sample size. For this analysis we randomly subsampled a group of 11 GFQ (same sample size as SFQ) and performed a new network analysis: the number of modules that we obtained was again higher in GFQ than in SFQ (90 modules vs. 8, respectively, data not shown) confirming that increased modularity in GFQ is not simply a consequence of larger sample size.

Results and Discussion

Comparative network analyses of GFQ vs. SFQ revealed that the two groups differ substantially (Fig. S8). GFQ were characterized by a complex network of 47 modules, including a large module of 6845 genes (53.5% of the total) and a remaining set of 46 smaller modules ranging from 11 to 2401 genes per module (median=29). The analysis of SFQ revealed a much simpler network composed of only 8 modules, where the largest accounted for 12647 genes (97.5% of the total) while the remaining modules ranged from 11 to 181 genes per module (median=30). Thus, modularity of gene networks in the brains of founding queens mirrors the complexity of the social environment that queens experience: the number of modules gets smaller as we move from the more complex social environment (group-founding) to the simpler one (single-founding). We found very good overlap between the two network analyses (Fig. S9): the genes included in the 6 smaller modules of the SFQ analyses were also part of GFQ modules (not including the large turquoise and grey modules). This indicates that similar genes respond to different social environments by redistributing themselves in different clusters.

Conclusions

With this analysis we show the complexity of the brain gene network increases in parallel with increasing complexity in the social environment. The higher modularity of the gene network in GFQ suggests that brain gene expression in these queens is more “compartmentalized” and less cohesive than in SFQ, where genes seem to work instead as a large single block. One possible explanation for this dissimilarity could be the occurrence of some sort of division of labour within incipient colonies of GFQ, whereby different queens engage in different tasks. Unfortunately, despite being well known for other ants that found colonies cooperatively (e.g., Ref.^11^), division of labour has not been reported in the literature for fire ants; it remains an intriguing hypothesis. Different tasks performed by individual GFQ could activate different groups of genes, or the same genes could respond differently to separate sets of tasks: this would result in an extremely complex array of interactions among genes that is captured by the high network modularity in GFQ. In contrast, all SFQ typically perform the same sets of tasks, as each of them has to take care of all activities that are essential for founding a new colony: this uniformity across queens is mirrored by the extremely cohesive brain gene expression network observed here, characterized by high gene-to-gene correlation and low modularity.

An alternative explanation could be that the higher network modularity in GFQ reflects the subtle dominance hierarchies that are present within social groups of founding queens. In this scenario, different modules could represent the molecular signatures of queens of different social status. Higher network modularity in GFQ also means lower connectivity overall among genes in their brain. This reinforces the idea that a large proportion of genes act independently of each other in these queens, probably responding in different ways to different cues due to internal social dynamics of founding groups.

References

1. Trapnell, C. *et al.* Transcript assembly and quantification by RNA-Seq reveals unannotated transcripts and isoform switching during cell differentiation. *Nature biotechnology* **28**, 511 (2010).

2. Soneson, C., Love, M. I. & Robinson, M. D. Differential analyses for RNA-seq: transcript-level estimates improve gene-level inferences. *F1000Research* **4**, (2015).

3. Love, M. I., Huber, W. & Anders, S. Moderated estimation of fold change and dispersion for RNA-seq data with DESeq2. *Genome biology* **15**, 1–21 (2014).

4. Manfredini, F. *et al.* Neurogenomic signatures of successes and failures in life-history transitions in a key insect pollinator. *Genome biology and evolution* **9**, 3059–3072 (2017).

5. Pracana, R. *et al.* Fire ant social chromosomes: Differences in number, sequence and expression of odorant binding proteins. *Evolution letters* **1**, 199–210 (2017).

6. Kanai, M. I., Okabe, M. & Hiromi, Y. seven-up Controls switching of transcription factors that specify temporal identities of Drosophila neuroblasts. *Developmental cell* **8**, 203–213 (2005).

7. Fahrbach, S. E. Structure of the mushroom bodies of the insect brain. *Annu. Rev. Entomol.* **51**, 209–232 (2006).

8. Tadros, W. *et al.* Dscam proteins direct dendritic targeting through adhesion. *Neuron* **89**, 480–493 (2016).

9. Wojtowicz, W. M., Flanagan, J. J., Millard, S. S., Zipursky, S. L. & Clemens, J. C. Alternative splicing of Drosophila Dscam generates axon guidance receptors that exhibit isoform-specific homophilic binding. *Cell* **118**, 619–633 (2004).

10. Rahman, M. M. *et al.* Search for phase specific genes in the brain of desert locust, Schistocerca gregaria (Orthoptera: Acrididae) by differential display polymerase chain reaction. *Comparative Biochemistry and Physiology Part A: Molecular & Integrative Physiology* **135**, 221–228 (2003).

11. Cahan, S. H. & Fewell, J. H. Division of labor and the evolution of task sharing in queen associations of the harvester ant Pogonomyrmex californicus. *Behavioral ecology and sociobiology* **56**, 9–17 (2004).


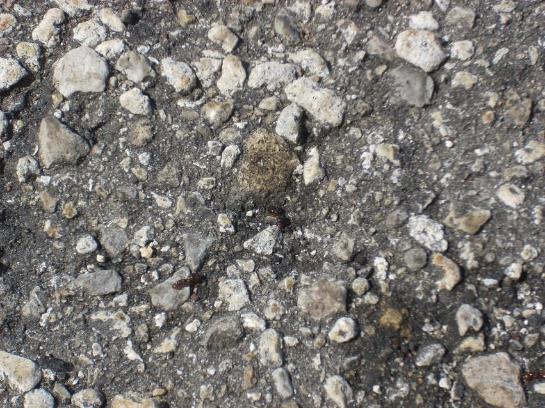

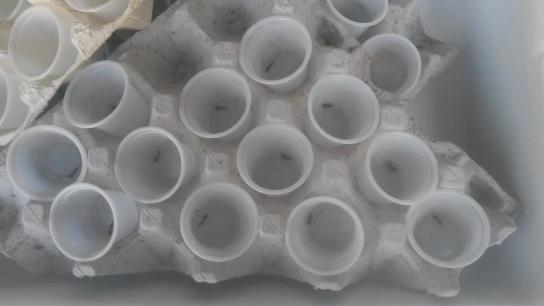


Fig. S1. Queens landed on the tarmac (left) and plastic cups used for sampling them (right).


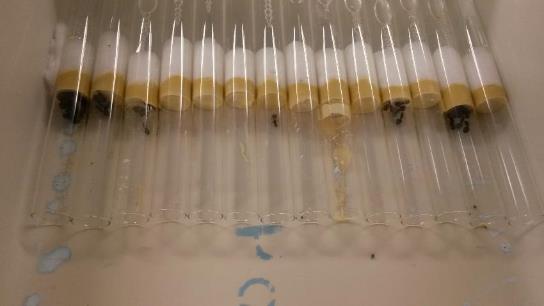


Fig. S2. Tray containing 14 nesting chambers.


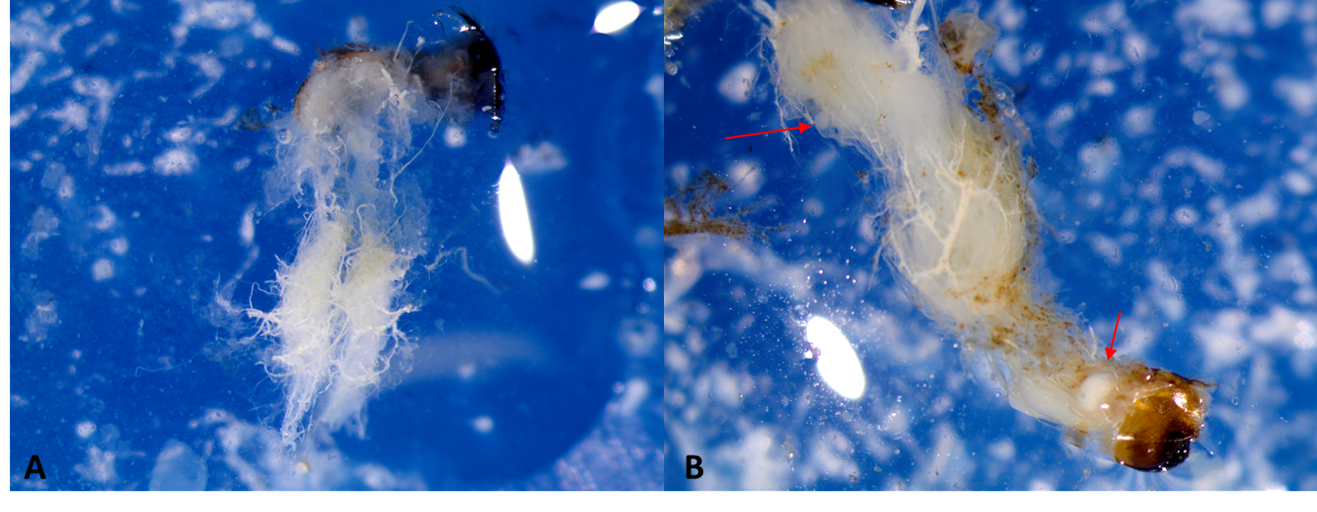

**C**

**Fig. S3**. Dissections of queens gasters: unmated queens showed reduced and filiform ovaries (A), while mated queens had developed ovaries (B) with visible eggs (red arrow) and visible spermatheca (white bean-shaped structure at the centre of the picture, panel C).

**
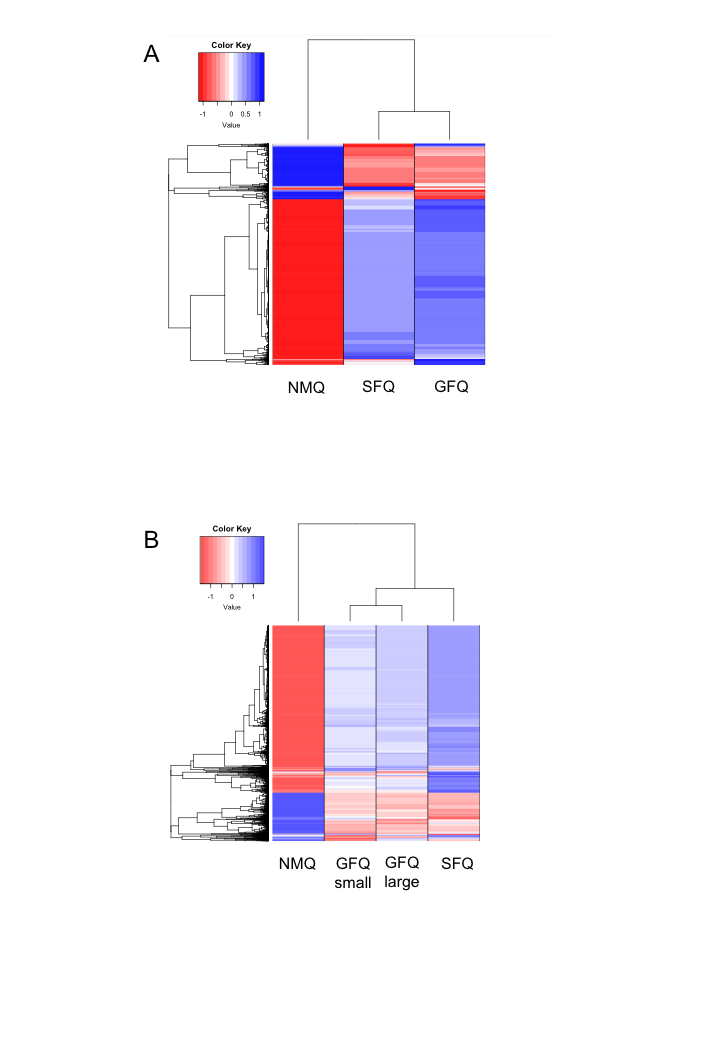
**

**Fig. S4**. Hierarchical clustering analyses by queen phenotype at 3dpf (A) and 25dpf (B). These analyses were performed on the sets of 2208 DEGs and 3256 DEGs, respectively, obtained with the LRT approach (FDR<0.001).

**Fig. S5**. Enrichment per module analysis to test whether any of the modules from the WGCNA approach had more supergene loci than expected by chance. One module (green) and the group of genes not included in any module (grey) were significantly enriched for supergene loci (Fisher Test, corrected p-value <0.05).

**
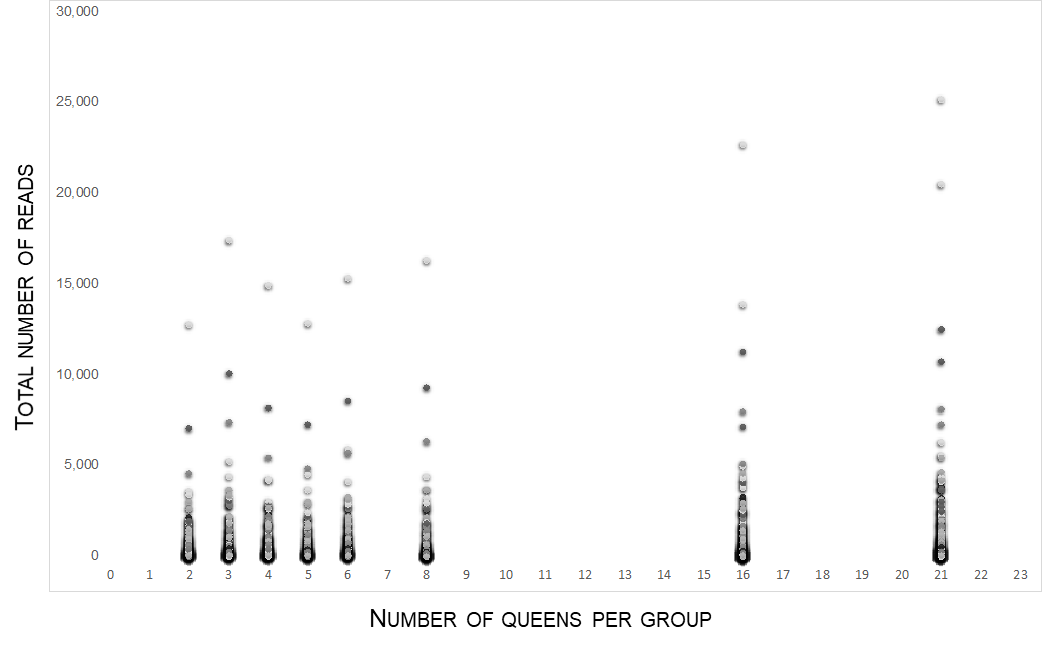
**

**Fig. S6**. Overview of read counts for the 258 genes that differed between GFQlarge and GFQsmall at FDR<0.05. Read counts are plotted according to the size of the group (number of queens within the group).

**Figure S7.** Gene expression analysis of large vs. small groups. Normalized read counts for 4 genes that differed between GFQlarge and GFQsmall and are particularly interesting for their role in cognition and neural functions. Read counts are plotted according to the 6 different groups of queens considered in this study.

GFQ

SFQ

534

639

711

2,401

6,845

12,647

181

132

149

>100

**Fig. S8.** Overall output for the comparative network analysis of group-founding vs. single-founding queens. Only modules (statistical cluster of genes that co-vary in expression) containing more than 100 genes are shown, with details on module ID (colour assigned by WGCNA analysis) and size (number of genes).

**Fig. S9.** Sankey diagram with the output of an Alluvial Plot analysis showing how genes in smaller SFQ modules cluster in GFQ modules (excluding large turquoise and grey modules).


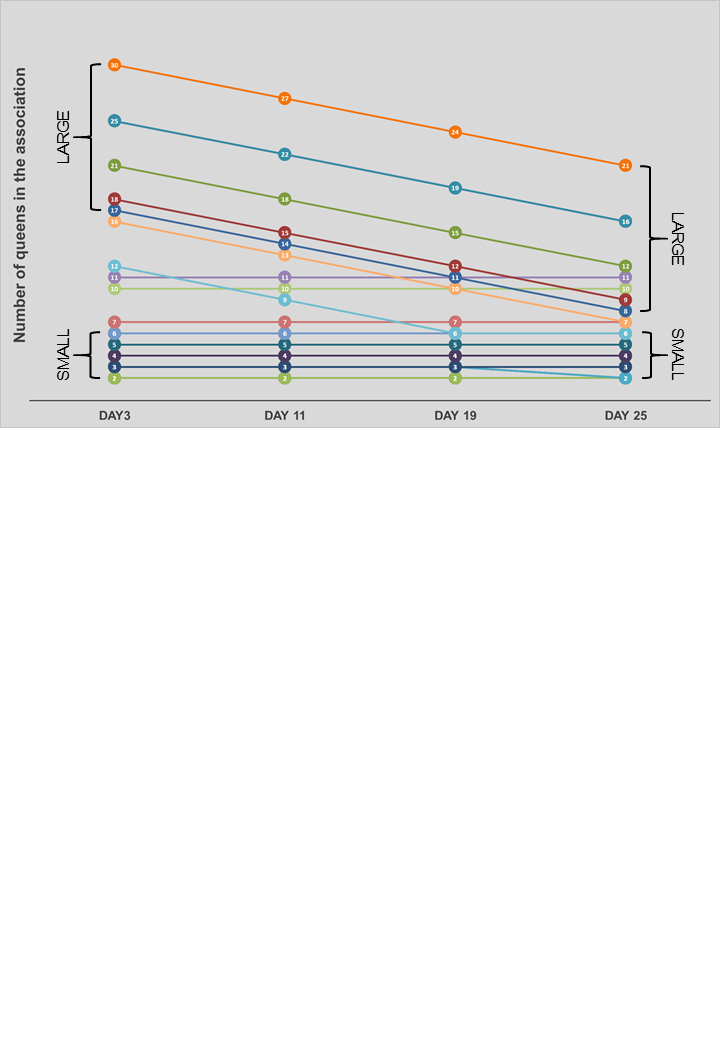


**Fig. S10**. Visualization of composition of GFQlarge and GFQsmall when sampled at 25dpf and also at previous time points. The picture also shows how group size changed over time as a consequence of natural queen mortality and also due to previous sampling at intermediate time points (day 11 and day 19, not used in this study).

A

B

**Fig. S11**. Examination of the effect of rearing tray on brain gene expression of founding queens. The two pictures show the two main Principal Components (PC) driving global gene expression in queens sampled at 3dpf (A) and 25dpf (B). Queens belonging to all SFQ and GFQ nests are color-coded according to the tray were they were housed (NMQ are not part of this analysis as they were not reared in trays). There is no evidence that tray might be a relevant factor for driving gene expression.

A

B

**Fig. S12**. Full overview of Principal Component analyses showing how much each PC contributed to variance of gene expression for queens sampled at 3dpf (A) and 25dpf (B).

**
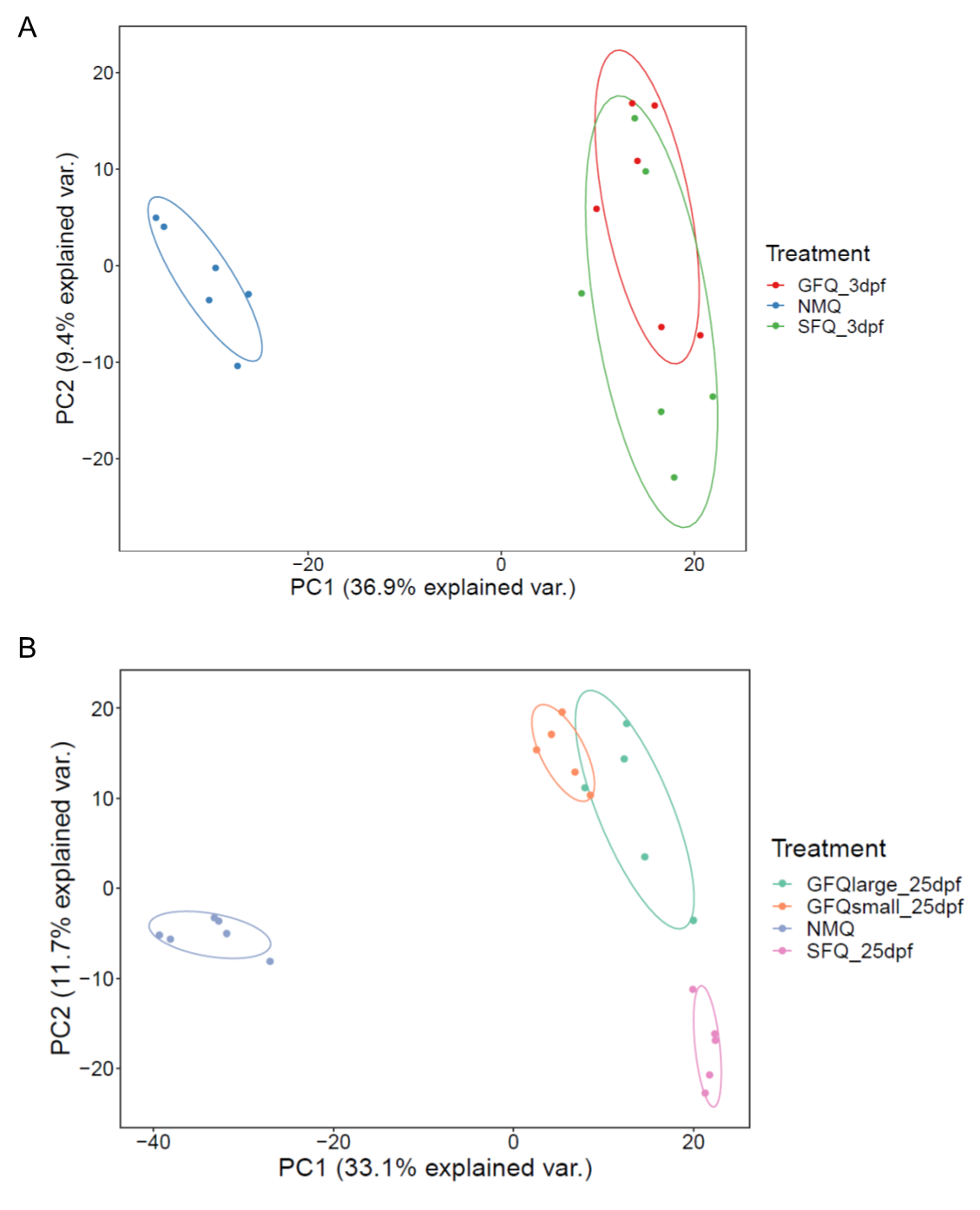
Fig. S13**. Visualization of the two main Principal Components (PCs) for PCA analyses at 3dpf (A) and 25dpf (B).

**Fig. S14**. Heatmap with the Euclidean distance between samples for all expression data (DESeq2 normalized and VST transformed), with labels for treatment and tray ID, for NMQs and queens sampled at 25dpf. Queens cluster according to treatment group while same tray IDs never cluster together.

A

B

**Fig. S15**. Comparison of overall genetic distance in expression within groups and among groups, for NMQs and queens sampled at 3dpf (A) and 25dpf (B). The median Euclidean distance based on all expression data (DESeq2 normalized and VST transformed) of each sample is compared to all samples in different groups, and samples are grouped by treatment. Distance of a sample to samples in its own group is smaller than that to all other groups.

| **Statistical approach** | **Focal comparison** | **Result** | **pvalue** | **adj_pvalue** |
| --- | --- | --- | --- | --- |
| LRT | 25dpf | enrichment | **0.049259076** | 0.098518153 |
| LRT | 3dpf | no_enrichment | 0.072138691 | 0.112215741 |
| pairwise_3dpf | NMQ_GFQ | no_enrichment | **0.007517841** | **0.030234891** |
| pairwise_3dpf | NMQ_SFQ | enrichment | 0.494485914 | 0.532523292 |
| pairwise_3dpf | NMQ_SFQ+GFQ | no_enrichment | 0.110149194 | 0.154208871 |
| pairwise_3dpf | SFQ_GFQ | enrichment | 0.1484538 | 0.1731961 |
| pairwise_25dpf | NMQ_GFQ | no_enrichment | 0.70715166 | 0.70715166 |
| pairwise_25dpf | NMQ_GFQlarge | no_enrichment | 0.145100796 | 0.1731961 |
| pairwise_25dpf | NMQ_GFQsmall | no_enrichment | 0.060739309 | 0.106293791 |
| pairwise_25dpf | NMQ_SFQ | enrichment | **0.030135105** | 0.070315245 |
| pairwise_25dpf | SFQ_GFQ | enrichment | **7.71E-06** | **6.55E-05** |
| pairwise_25dpf | SFQ_GFQlarge | enrichment | **0.00863854** | **0.030234891** |
| pairwise_25dpf | SFQ_GFQsmall | enrichment | **9.36E-06** | **6.55E-05** |
| pairwise_25dpf | GFQlarge_GFQsmall | no_enrichment | **0.021883859** | 0.061274804 |

**Table S1**. Enrichment analysis for differentially expressed genes. A significant enrichment for supergene loci was detected among comparisons at 25dpf, in particular between SFQ and both GFQlarge and GFQsmall (Kolmogorov-Smirnoff Test, corrected p-value<0.05).

Other supporting information for this manuscript:

Dataset S1 (separate file). Sample details for RNAseq experiments. Details on ant queens selected for RNAseq, and quantities and quality of RNA samples from queens’ brains that were used to prepare libraries. Only samples labelled in red were used in the end.

Dataset S2 (separate file). Pairwise comparisons at 3dpf. Details are provided about gene expression analyses (fold-change differences, p-values and corrected p-values for all genes analyzed) and Gene Ontology analyses (GO terms overrepresented at corrected p-values<0.05) for all pairwise comparisons.

Dataset S3 (separate file). Pairwise comparisons at 25dpf. Details are provided about gene expression analyses (fold-change differences, p-values and corrected p-values for all genes analyzed) and Gene Ontology analyses (GO terms overrepresented at corrected p-values<0.05) for all pairwise comparisons.

Dataset S4 (separate file). WGCNA modules significantly associated with phenotypes of interest. Details are provided of the gene to gene correlation for all genes included in the module.

Dataset S5 (separate file). Eigenvalues and loadings for all PCs obtained at the two timepoints.
